# Sinonasal Anatomic Variations Across Vertical Skeletal Patterns in Skeletal Class I Adults: A Retrospective Cone-Beam Computed Tomography Study

**DOI:** 10.3390/diagnostics16213534

**Published:** 2026-10-28

**Authors:** Samet Özden, Beril Erdil, Elif Sobi, Numan Dedeoğlu

**Affiliations:** 1 Department of Orthodontics, Faculty of Dentistry, İnönü University, Malatya 44280, Türkiye; beril.erdil@inonu.edu.tr; 2 Department of Oral and Maxillofacial Radiology, Faculty of Dentistry, İnönü University, Malatya 44280, Türkiye; elif.yildiz@inonu.edu.tr (E.S.); numan.dedeoglu@inonu.edu.tr (N.D.)

**Keywords:** cone-beam computed tomography, paranasal sinuses, nasal cavity, anatomic variation, vertical skeletal pattern, orthodontics

## Abstract

**Background/Objectives:** Vertical skeletal morphology may influence maxillofacial dimensions, but its relationship with discrete sinonasal anatomic variations remains uncertain. This study compared the prevalence of paranasal sinus and nasal cavity variations among adults with hypodivergent, hyperdivergent, and normodivergent skeletal patterns. **Methods:** Faculty archives spanning 2016–2025 were screened. Eighty-seven untreated adults with a skeletal Class I relationship were included and categorized according to their vertical skeletal pattern as follows: 27 hypodivergent, 26 hyperdivergent, and 34 normodivergent. All cone-beam computed tomography (CBCT) examinations were acquired using a single NewTom 5G unit with 0.25 mm isotropic voxels. Nasal septal deviation and septal spur were evaluated per participant; 22 additional variants were evaluated bilaterally. Side-level comparisons used binary logistic generalized estimating equations with Benjamini–Hochberg false-discovery-rate correction. **Results:** Age (*p* = 0.5072), nasal septal deviation (*p* = 0.2247), and septal spur (*p* = 0.9294) did not differ significantly among groups. The paradoxical middle turbinate showed a nominal group effect (Wald χ^2^ = 6.199, *p* = 0.0451), but the association was no longer significant after false discovery rate correction (q = 0.4642). No other estimable side-level group or within-group right–left comparison was significant. **Conclusions:** No statistically significant associations were detected between vertical skeletal pattern and sinonasal anatomical variations in this sample of skeletal Class I adults. However, small-to-moderate associations cannot be excluded, particularly for rare variants. The nominal finding for the paradoxical middle turbinate requires confirmation in larger, prospectively defined cohorts.

## 1. Introduction

The maxillary sinus is the largest paranasal sinus and develops in close relation to the surrounding maxillofacial skeleton. Although most postnatal enlargement occurs during childhood and adolescence, its morphology varies considerably among adults. Craniofacial dimensions and vertical skeletal pattern have been associated with maxillary sinus volume, linear dimensions, and the relationship between the sinus floor and posterior tooth roots [1,2,3,4,5]. Furthermore, a CBCT study of skeletal Class I and III adults identified associations between jaw volumes and cephalometric measurements, including greater mandibular volume in hypodivergent than hyperdivergent Class III participants [6].

This relationship has practical relevance in orthodontics. The maxillary sinus borders the posterior dentition and alveolar process, and its morphology may influence posterior tooth movement, molar uprighting, skeletal anchorage, maxillary expansion, and other interventions involving the posterior maxilla. Identification of the sinus floor and adjacent cortical boundaries may help prevent unintended sinus penetration during temporary anchorage device placement and define the available three-dimensional path for tooth movement [4,5,7].

Sinonasal assessment also extends beyond the dentoalveolar region. Variations involving the nasal septum, turbinates, ethmoid roof, sinus ostia, sphenoid sinus, optic canal, vidian canal, maxillary nerve canal, and carotid canal can alter drainage pathways or the spatial relationships between critical neurovascular structures and surgical corridors. Their reported prevalence varies among populations and imaging protocols, making explicit operational definitions essential for comparing prevalence estimates [8,9,10].

Cone-beam computed tomography (CBCT) provides multiplanar visualization of osseous maxillofacial anatomy and can demonstrate many sinonasal variants when the region is included in an examination acquired for a justified dental or maxillofacial indication [9,11]. CBCT is not a substitute for indication-specific medical CT, magnetic resonance imaging, nasal endoscopy, or functional testing when soft-tissue disease or airway physiology is the primary concern. Its value in retrospective orthodontic research lies in its ability to evaluate bilateral structures without the superimposition inherent to two-dimensional imaging.

Existing studies relating craniofacial pattern to the paranasal sinuses have investigated sinus dimensions [2,3], sinonasal variants and sinus volume across sagittal skeletal classes [12], and paranasal variants in children [13]. Studies comparing vertical facial patterns have primarily examined sinus volume or linear dimensions [14,15], leaving the distribution of a broader range of discrete sinonasal variants less clearly characterized. Therefore, this study compared participant-level and side-level sinonasal variations among untreated adults with hypodivergent, hyperdivergent, and normodivergent skeletal patterns while restricting sagittal and dental relationships to Class I. Between-group and within-group right–left comparisons were exploratory.

## 2. Materials and Methods

### 2.1. Study Design, Setting, and Ethics

This retrospective cross-sectional study used CBCT examinations acquired between 2016 and 2025 and stored in the archive of the Department of Oral and Maxillofacial Radiology, Faculty of Dentistry, İnönü University. A total of 1789 patient records were screened. Of these, 1123 records were excluded because of craniofacial anomalies, previous orthodontic treatment, severe inflammatory or other sinonasal disease, previous head-and-neck trauma, previous sinonasal surgery, inadequate field of view, image artifacts, or insufficient diagnostic quality. Among the remaining 666 records, 579 did not satisfy the prespecified age, sagittal skeletal, dental, or vertical skeletal-pattern eligibility criteria. The final sample comprised 87 participants. Smoking history and allergic rhinitis status could not be retrieved from the archived records.

The İnönü University Non-Interventional Clinical Research Ethics Committee approved the study on 21 August 2025 (decision no. 2025/8169). Signed consent forms obtained during the patients’ original clinical care are retained in the faculty archive. Because the study involved retrospective analysis of pre-existing anonymized examinations, the ethics committee waived the requirement for additional study-specific individual informed consent. The study was conducted in accordance with the Declaration of Helsinki.

### 2.2. Eligibility and Skeletal-Pattern Classification

Eligibility required an age of 18–30 years, a diagnostic-quality CBCT examination covering the complete region of interest, no history of orthodontic treatment, a bilateral Angle Class I molar relationship, and a skeletal Class I sagittal relationship. Skeletal Class I status required both an A–nasion–B (ANB) angle of 0°–4° and a Wits appraisal of −2 to +2 mm. Wits appraisal was recorded as the linear AO–BO distance on the functional occlusal plane [16].

Vertical skeletal pattern was determined from the GoGn–SN angle and Frankfort–mandibular plane angle (FMA). Hypodivergent classification required GoGn–SN < 32° and FMA < 25°; normodivergent classification required GoGn–SN of 32°–38° and FMA of 25°–29°; and hyperdivergent classification required GoGn–SN > 38° and FMA > 29°. Only records in which both measurements indicated the same vertical category were allocated to a group. ANB, Wits appraisal, GoGn–SN, and FMA were measured on the patients’ lateral cephalometric radiographs by an experienced orthodontist using manual landmark identification and tracing with the NemoCeph module of NemoStudio version 25.0.0.0 (Nemotec S.L., Madrid, Spain). Cephalometric tracing and landmark identification were performed with the examiner blinded to the sinonasal findings.

Exclusion criteria were a craniofacial anomaly; previous orthodontic treatment; previous head-and-neck trauma or sinonasal surgery; an image artifact, inadequate field of view, or insufficient diagnostic quality that prevented evaluation; and severe inflammatory or other sinonasal disease. Severe inflammatory disease was defined radiographically as complete or near-complete sinus opacification, an air–fluid level, diffuse polypoid mucosal disease obscuring sinus boundaries or drainage pathways, or an expansile or destructive inflammatory lesion [17,18]. Localized mucosal thickening and dome-shaped mucous retention cysts were not grounds for exclusion because they were evaluated as study variables.

The final sample included 27 hypodivergent participants (18 women and 9 men), 26 hyperdivergent participants (18 women and 8 men), and 34 normodivergent participants (20 women and 14 men). Because all participants were adults, the term vertical skeletal pattern is used throughout rather than growth pattern.

### 2.3. Sample Size

An a priori power analysis was performed with G*Power version 3.1.9.7. The calculation was based on the prevalence of concha bullosa reported across three sagittal skeletal-malocclusion groups in a pediatric CBCT study [13]. The published group prevalences yielded Cohen’s w = 0.450 (unrounded value: 0.44957). For a 2 × 3 chi-square test with two degrees of freedom, α = 0.05, and 85% power, the minimum total sample was 55. With equal allocation, this value was rounded upward to 19 participants per group (57 participants in total). The final sample of 87 participants exceeded this requirement [19]. This calculation addressed the assumed concha bullosa effect and did not establish adequate power for small-to-moderate differences, rare variants, or all clustered comparisons after adjustment for multiple testing.

### 2.4. CBCT Acquisition and Image Review

All 87 examinations were acquired using the same flat-panel NewTom 5G CBCT unit (QR S.r.l., Verona, Italy) using 110 kVp, 1–20 mA, an 18 s scan time, and a 3.6 s exposure time. The field of view (height × diameter) was 15 × 12 cm. Datasets were transferred to NNT software (NewTom, Verona, Italy), reconstructed at 0.25 mm intervals, and evaluated in axial, coronal, and sagittal planes. Window level, contrast, magnification, and multiplanar orientation were adjusted when necessary for each structure. Because one scanner and one nominal acquisition and reconstruction protocol were used for all examinations, scanner distribution could not differ among groups, and no protocol-harmonization procedure was required.

The examinations had originally been acquired for clinical purposes independent of this study. In the hypodivergent group (*n* = 27), clinical indications comprised evaluation of impacted teeth (*n* = 21; predominantly mandibular; 2 patients with maxillary third molars without direct sinus contact), evaluation of pathology (*n* = 2), evaluation of miniscrew insertion sites before appliance design (*n* = 2), and assessment before surgically assisted rapid maxillary expansion (SARME; *n* = 2). In the hyperdivergent group (*n* = 26), indications comprised evaluation of impacted teeth (*n* = 20; predominantly mandibular; 1 patient with maxillary third molars without direct sinus contact), evaluation of pathology (*n* = 4), and assessment before SARME (*n* = 1); the indication was unavailable for one participant. In the normodivergent group (*n* = 34), indications comprised evaluation of impacted teeth (*n* = 25; predominantly mandibular; 2 patients with maxillary third molars without direct sinus contact), evaluation of pathology (*n* = 2), evaluation of miniscrew insertion sites before appliance design (*n* = 4), and assessment before SARME (*n* = 1); indications were unavailable for two participants. No examination was acquired solely for research. The region of interest extended horizontally from the anterior wall of the frontal sinus to the posterior boundary of the sphenoid sinus and vertically from the floor of the maxillary sinuses to the superior boundary of the nasal cavity.

### 2.5. Image Assessment and Operational Definitions

**Figure 1:**
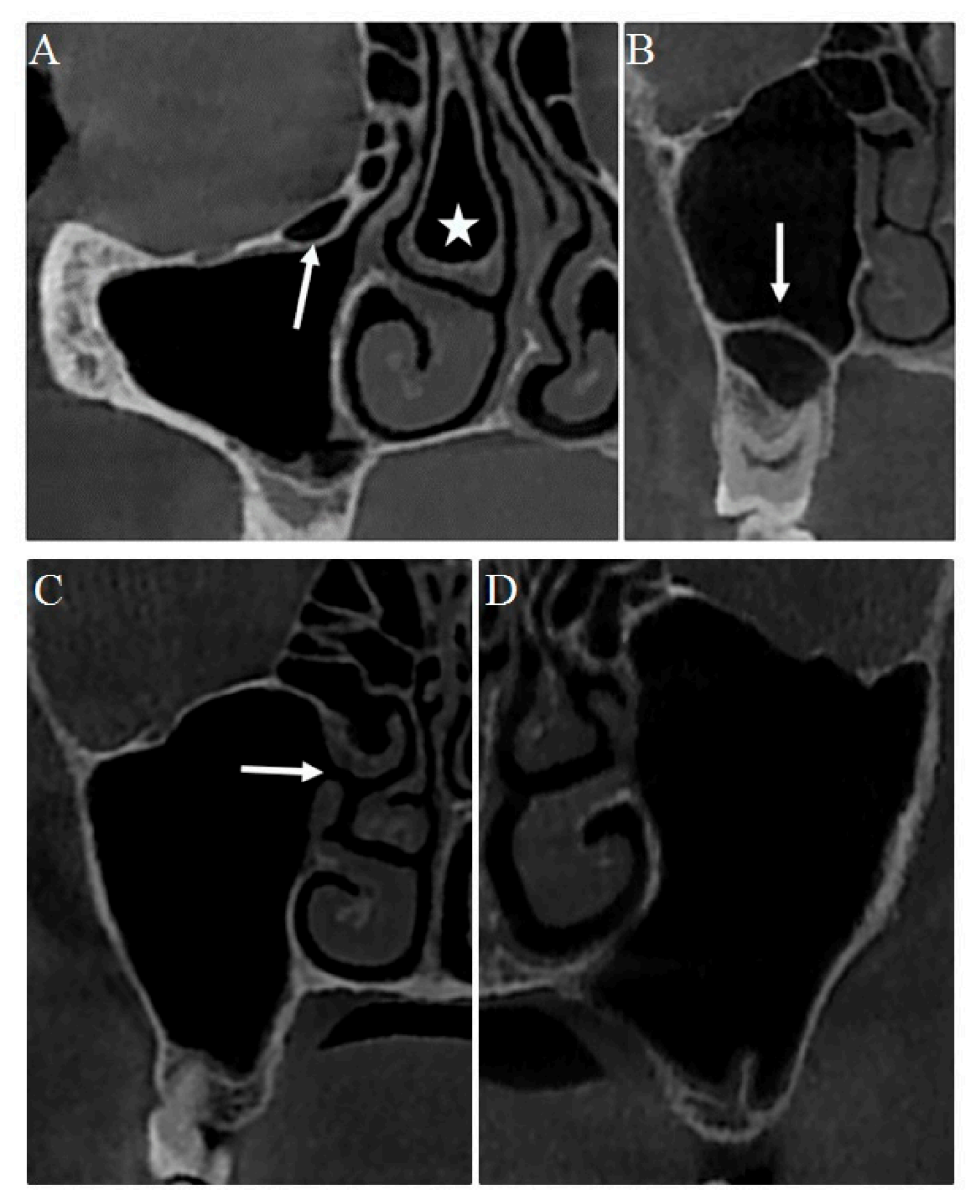
Coronal cone-beam computed tomography images demonstrate (**A**) a Haller cell (arrow) and concha bullosa (asterisk); (**B**) a maxillary sinus septum (arrow); (**C**) an accessory maxillary ostium (arrow); and (**D**) extensive maxillary sinus pneumatization extending into the alveolar process and zygomatic bone.

**Figure 2:**
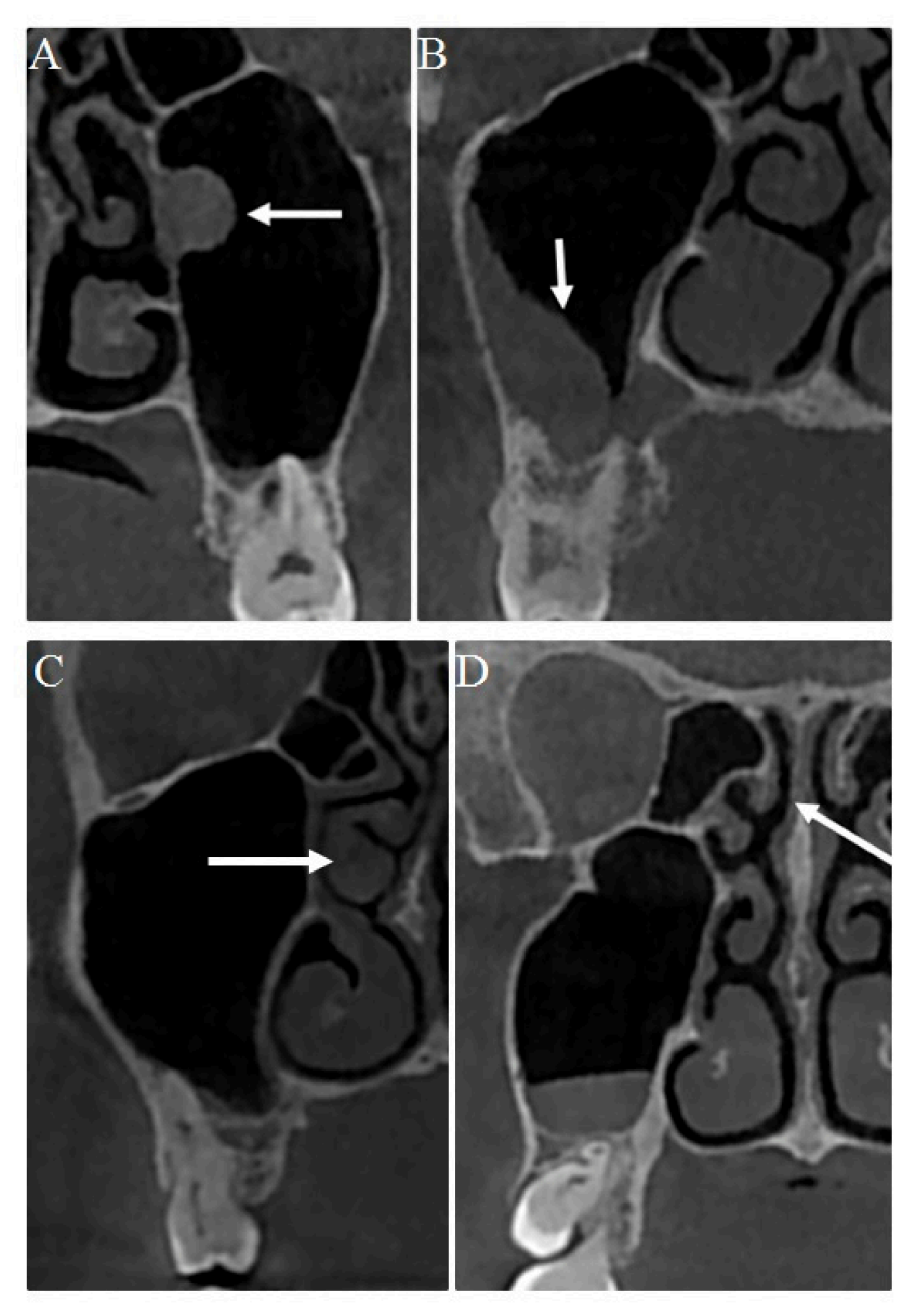
Coronal cone-beam computed tomography images demonstrate (**A**) a mucous retention cyst (arrow); (**B**) maxillary sinus mucosal thickening (arrow); (**C**) a paradoxical middle turbinate (arrow); and (**D**) a supreme turbinate (arrow).

**Figure 3:**
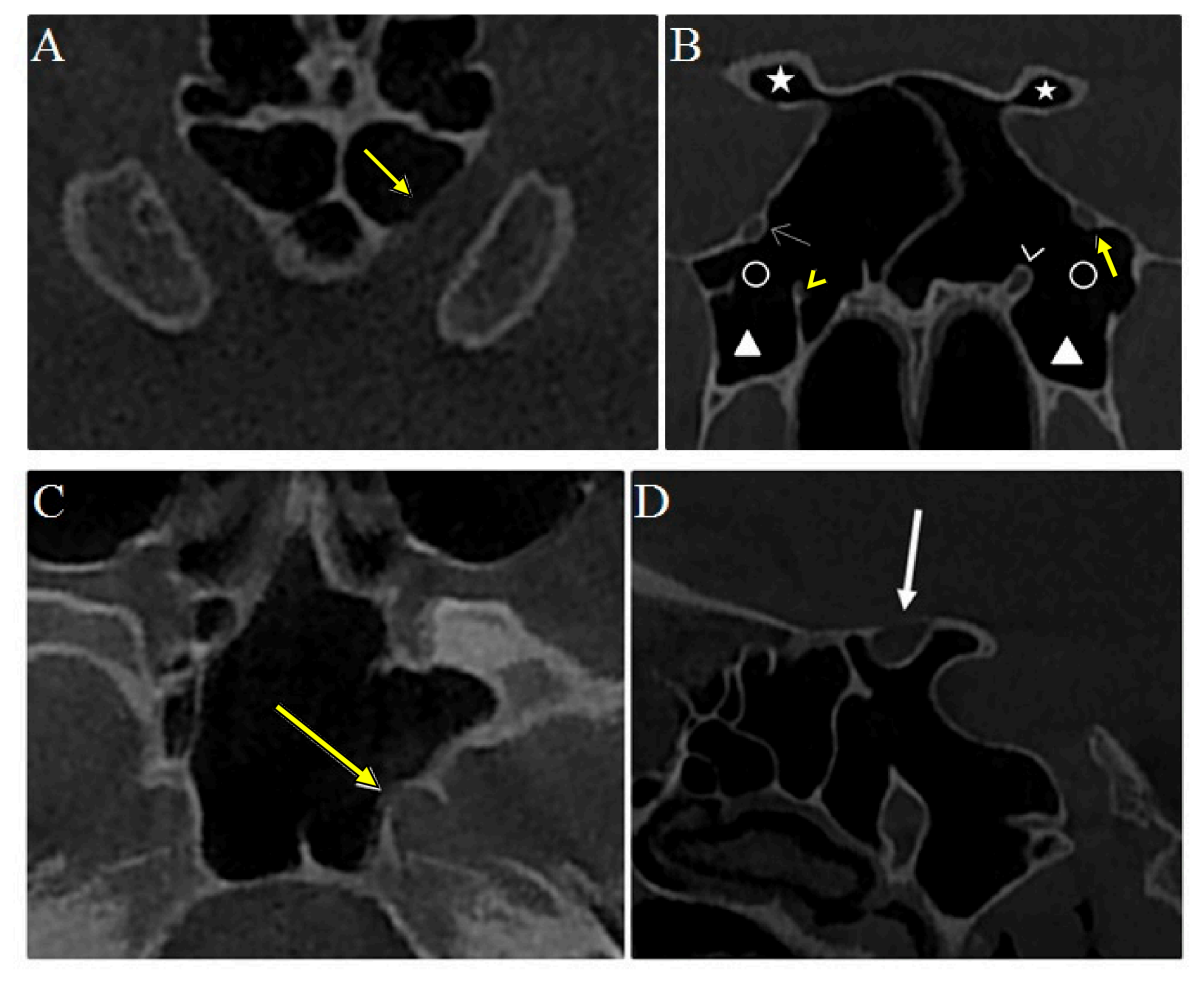
Cone-beam computed tomography images demonstrate (**A**) optic canal dehiscence on an axial image (yellow-highlighted arrow); (**B**) vidian canal dehiscence (yellow-highlighted arrowhead on the left), maxillary nerve canal dehiscence (yellow-highlighted arrow on the right), vidian canal protrusion (white arrowhead on the right), maxillary nerve canal protrusion (white arrow on the left), anterior clinoid process pneumatization (white asterisks), greater wing pneumatization (white circles), and pterygoid process pneumatization (white triangles) on a coronal image; (**C**) carotid canal dehiscence on an axial image (yellow-highlighted arrow); and (**D**) optic canal protrusion on a sagittal image (white arrow).

**Figure 4:**
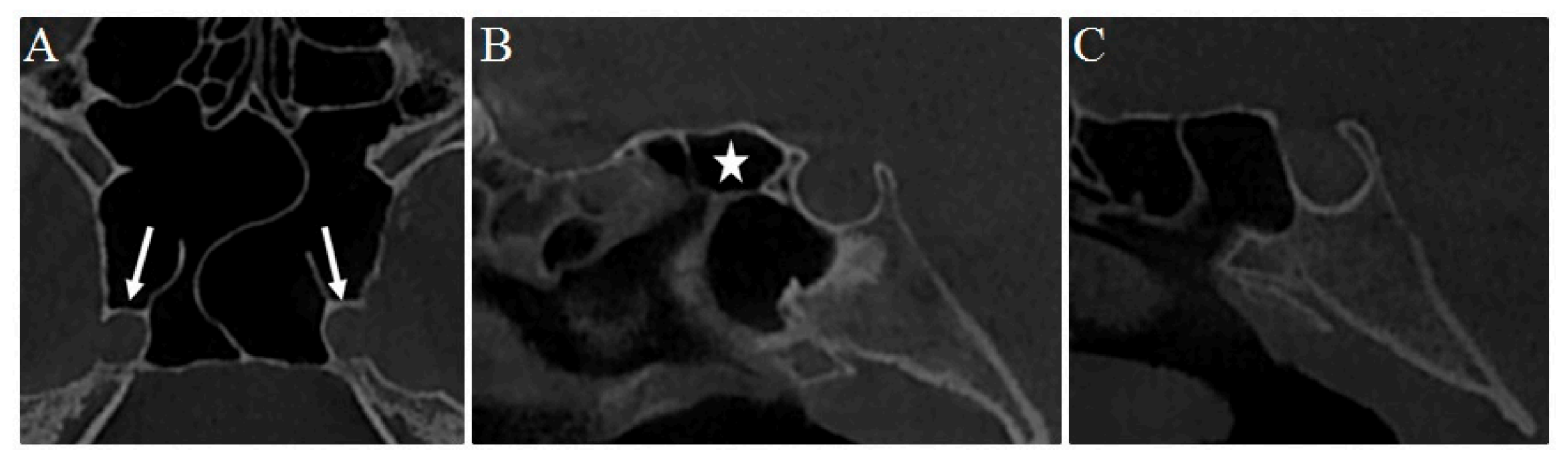
Cone-beam computed tomography images demonstrate (**A**) carotid canal protrusion on an axial image (arrows); (**B**) an Onodi cell on a sagittal image (asterisk); and (**C**) sphenoid sinus hypoplasia on a sagittal image.

The primary CBCT assessments were performed by an oral and maxillofacial radiologist (N.D.) with 15 years of CBCT experience. Nasal septal deviation and septal spur were recorded once per participant. The remaining 22 findings were recorded separately on the right and left sides. The term maxillary nerve canal refers to the V2/foramen rotundum corridor. Operational definitions are presented in Table 1. Representative CBCT appearances of the nasal-cavity and maxillary-sinus findings are shown in Figure 1 and Figure 2, and representative sphenoid-region and neurovascular-canal findings are shown in Figure 3 and Figure 4.

Binary classification was used to compare the prevalence of findings meeting the operational definitions in Table 1. The >4 mm threshold for nasal septal deviation follows the definition of Smith et al. [20]. The >3.0 mm threshold for mucosal thickening has also been used in previous CBCT research [21]. It provides a more restrictive classification than >2.0 mm by excluding thicknesses of >2.0 to ≤3.0 mm from the positive category. These cutoffs were used as operational thresholds for prevalence estimation, not as stand-alone criteria for clinical disease. Extensive maxillary sinus pneumatization was classified using the combined alveolar–zygomatic extent criterion in Table 1; these anatomical extensions have been assessed in previous CBCT studies [22,23]. Sinus volume was not measured. Only binary classifications were recorded for these findings.

**Table 1:** Operational definitions of the evaluated findings.

Finding	Operational Definition
Nasal septal deviation	Displacement of the nasal septum by >4 mm from the midline on coronal CBCT sections, measured at the point of maximum deviation [20].
Septal spur	A focal sharp angulation or bony projection arising from the nasal septum, typically at the junction of the perpendicular plate of the ethmoid and the superior surface of the vomer, irrespective of size [24].
Haller cell	An ethmoid air cell located in the medial portion of the orbital floor and along the medial roof of the maxillary sinus [25].
Maxillary sinus septum	A thin cortical bony partition arising from the sinus floor and subdividing the sinus cavity [26].
Accessory maxillary ostium	An additional opening distinct from the natural maxillary ostium [27].
Extensive maxillary sinus pneumatization	Pneumatization of the same maxillary sinus extending into both the alveolar process and zygomatic bone without osseous erosion [22,23].
Mucous retention cyst	A smooth, dome-shaped soft-tissue opacity arising from a maxillary sinus wall [18,28,29].
Maxillary sinus mucosal thickening	Mucosal thickness > 3.0 mm along the sinus wall [21].
Paradoxical middle turbinate	A middle turbinate whose convexity is directed toward the nasal septum [9,10].
Concha bullosa	For this study, concha bullosa was defined as pneumatization of the middle turbinate; pneumatization of other turbinates was not counted [20,30,31].
Supreme turbinate	An additional turbinate arising from the medial ethmoid labyrinth superior to the superior turbinate [32].
Canal protrusion	Any degree of protrusion of the optic, vidian, maxillary nerve, or carotid canal into the sphenoid sinus [33,34,35].
Canal dehiscence	Complete loss of bony cortical continuity surrounding the specified canal, confirmed in at least two orthogonal planes. Cortical thinning or an apparent defect visible in only one plane was not classified as dehiscence.
Anterior clinoid process pneumatization	Extension of the sphenoid sinus cavity into the anterior clinoid process [33,34,35].
Greater wing pneumatization	Horizontal extension of sphenoid sinus pneumatization beyond the vidian canal [33,34,35,36].
Pterygoid process pneumatization	Extension of sphenoid sinus pneumatization beyond the vertical plane of the foramen rotundum [33,34,35,36].
Onodi cell	The most posterior ethmoid cell extending superolaterally to the sphenoid sinus [37].
Sphenoid sinus hypoplasia	Pneumatization ending anterior to the vertical plane through the tuberculum sellae (presphenoid pattern) [38].

### 2.6. Intraobserver and Interobserver Reliability

All 87 CBCT examinations were reevaluated by the same oral and maxillofacial radiologist after a 1-month interval. The observer was blinded to the initial scores. Agreement was assessed separately for each finding using percentage agreement and unweighted Cohen’s kappa, with 87 paired ratings for each participant-level finding and 174 paired side-level ratings for each bilateral finding. Agreement ranged from 98.85% to 100%, and Cohen’s κ ranged from 0.971 to 1.000.

A second oral and maxillofacial radiologist (E.S.) independently assessed all 87 examinations using the same definitions, blinded to the first reader’s scores and vertical skeletal groups. Interobserver agreement was calculated separately for each finding using percentage agreement and unweighted Cohen’s kappa (87 paired participant-level or 174 paired side-level ratings). Agreement ranged from 87.4% to 100%, with κ = 0.875–0.999.

### 2.7. Statistical Analysis

Statistical analyses were performed using IBM SPSS Statistics version 26.0 (IBM Corp., Armonk, NY, USA). The age distribution was assessed graphically and with the Shapiro–Wilk test. Continuous data are summarized using the mean and standard deviation and the median and range, as appropriate; categorical data are reported as number and percentage. Age was compared among groups with the Kruskal–Wallis test. Sex, nasal septal deviation, and septal spur were compared with Pearson’s chi-square test.

For each bilateral finding, a separate binary logistic generalized estimating equation (GEE) model was fitted with the finding (present/absent) as the dependent variable, vertical skeletal-pattern group as a three-level between-participant factor, participant identifier as the clustering variable, and side as the repeated within-participant factor. Each model included an intercept and two group-indicator coefficients and used 87 independent participant clusters. The models used a binomial distribution, logit link, exchangeable working correlation matrix, and empirical robust standard errors. The omnibus group effect was evaluated with the Wald chi-square statistic. Model-derived pairwise odds ratios (ORs) with 95% confidence intervals (CIs) were reported as secondary effect-size estimates. When zero-event groups caused separation, the omnibus group comparison was reported as not estimable (NE). Pairwise ORs and CIs were not reported for outcomes whose full three-group GEE model did not yield a complete set of finite group-effect estimates; no continuity correction was applied. Right–left frequencies within each group were compared with the exact McNemar test. Two-sided *p* < 0.05 was the nominal significance threshold. The estimable side-level omnibus tests were additionally evaluated with the Benjamini–Hochberg false-discovery-rate (FDR) procedure, with q < 0.05 indicating evidence robust to multiple testing [39].

Participant-level prevalences of carotid canal dehiscence, sphenoid sinus hypoplasia, and optic canal dehiscence were additionally summarized with two-sided exact Clopper–Pearson 95% CIs, defining a participant as affected when the finding was present on either or both sides. As a sensitivity analysis, all 20 estimable bilateral GEE models were re-evaluated using the Mancl–DeRouen bias-corrected sandwich covariance estimator, retaining the original model specifications, two-degree-of-freedom omnibus Wald tests, and Benjamini–Hochberg correction across the same 20 outcomes [40].

Additional analyses adjusted for sex by adding a female-versus-male indicator to each vertical-group model. Bilateral models retained participant clustering, the binomial logit link, exchangeable working correlation, and robust covariance. Participant-level septal findings were assessed with sex-adjusted logistic regression and robust covariance. Complete or quasi-complete separation was checked before reporting estimates. FDR correction was applied separately to the 19 estimable bilateral group tests and 19 sex-coefficient tests; participant-level tests remained separate. Adjusted group ORs and 95% CIs are reported in Table S5, and sex coefficients in Table S6.

## 3. Results

### 3.1. Study Selection and Participant Characteristics

Of the 1789 archive records screened, 1123 were excluded according to the predefined exclusion criteria. Of the remaining 666 records, 579 did not meet the complete inclusion criteria, leaving 87 participants for analysis. Median ages were 21 years in the hypodivergent and hyperdivergent groups and 20 years in the normodivergent group; age did not differ significantly among groups (H = 1.358, *p* = 0.5072). Sex distribution also did not differ significantly (*p* = 0.6750; Table 2).

### 3.2. Participant-Level Nasal Septal Findings

Nasal septal deviation was present in 88.9% of hypodivergent, 76.9% of hyperdivergent, and 70.6% of normodivergent participants. Septal spurs were present in 37.0%, 34.6%, and 32.4%, respectively. Neither finding differed significantly among groups (Table 3).

### 3.3. Side-Level Anatomic Variations

The side-level analysis included 54 hypodivergent, 52 hyperdivergent, and 68 normodivergent sides. Paradoxical middle turbinate showed a nominal omnibus group effect (Wald χ^2^ = 6.199, *p* = 0.0451), but it did not remain significant after FDR correction (q = 0.4642). No other estimable omnibus GEE effect reached *p* < 0.05. No affected sides were observed for carotid canal dehiscence in the hyperdivergent group or for sphenoid sinus hypoplasia in the hypodivergent and normodivergent groups. These zero-event cells caused separation in the specified GEE models, preventing valid omnibus group comparisons. Consequently, no omnibus GEE *p*-values or FDR-adjusted q-values were reported for these two outcomes. They are reported as not estimable (NE) and should not be interpreted as nonsignificant between-group comparisons. Table 4 presents the final omnibus results, and model-derived pairwise ORs with 95% CIs are provided in Table S4.

At the participant level, carotid canal dehiscence was present in 2/87 participants (2.30%; exact 95% CI, 0.28–8.06%), sphenoid sinus hypoplasia in 1/87 (1.15%; 0.03–6.24%), and optic canal dehiscence in 5/87 (5.75%; 1.89–12.90%). The absence of carotid canal dehiscence among the 26 hyperdivergent participants corresponded to an exact 95% CI of 0.00–13.23%. These participant-level estimates describe findings on at least one side.

The Mancl–DeRouen sensitivity analysis produced no statistically significant group comparisons among the 20 estimable outcomes (minimum unadjusted *p* = 0.05602; minimum FDR-adjusted q = 0.54374). The absence of FDR-significant findings was therefore unchanged after the covariance correction.

After adjustment for sex, none of the 19 estimable bilateral group comparisons survived FDR correction (minimum *p* = 0.05003; minimum q = 0.4073; Table S5). The adjusted group *p*-values were 0.2622 for nasal septal deviation and 0.9103 for septal spur. Optic canal dehiscence occurred only in men, causing separation after sex adjustment; carotid canal dehiscence and sphenoid sinus hypoplasia also remained non-estimable. No sex coefficient survived its separate FDR correction (minimum q = 0.05574; Table S6).

### 3.4. Right–Left Comparisons

No exact McNemar comparison between the right and left sides reached *p* < 0.05 in any vertical skeletal-pattern group (Tables S1–S3). The largest observed asymmetry involved maxillary nerve canal dehiscence in the hyperdivergent group (right, 3.8%; left, 23.1%; *p* = 0.0625), but this difference was not significant.

## 4. Discussion

Among findings with estimable group comparisons, no statistically significant association between vertical skeletal pattern and the evaluated sinonasal variations was detected after correction for multiple testing, including in the sex-adjusted analyses. The nominal finding for paradoxical middle turbinate did not survive FDR correction. These nonsignificant findings do not establish biological independence or anatomical equivalence. Interpretation therefore emphasizes the observed prevalences and the precision of the association estimates, consistent with STROBE guidance for studies using existing data [41].

A developmental association between facial morphology and the paranasal sinuses remains biologically plausible. The maxillary sinus enlarges within the maxilla and shares boundaries with the nasal cavity, orbital floor, alveolar process, and posterior teeth. Differences in vertical facial proportions can coexist with differences in posterior maxillary height, transverse width, dentoalveolar compensation, and sinus dimensions. Several CBCT studies have reported differences in maxillary sinus volume or linear measurements among high-, low-, and average-angle groups, but the direction and magnitude of these associations have been inconsistent [2,3,14,15]. Systematic evidence is also limited by inconsistent control of age, sex, and skeletal growth pattern [2]. Thus, an association with sinus size does not necessarily extend to discrete anatomic variants of the sinonasal region.

This distinction between a continuous dimension and a discrete morphologic variant helps interpret the predominantly nonsignificant group comparisons. Sinus volume reflects the combined effects of maxillary growth and pneumatization over time. In contrast, Haller cells, accessory ostia, sinus septa, turbinate pneumatization, and sphenoid extensions arise through partly distinct developmental pathways and may be influenced by local anatomy, population background, and observer-applied definitions. Previous investigations of septal deviation, concha bullosa, dental status, and other sinonasal findings have reported variable relationships with maxillary sinus volume [12,42,43,44,45]. These findings support the possibility of selective associations, but do not demonstrate that a vertical skeletal category reliably predicts the presence of individual variants.

The present study assessed categorical sinonasal variants. Cortical plate thickness, alveolar ridge height, root-to-sinus floor distances, and miniscrew insertion-site dimensions were not measured. In particular, the categorical designation of extensive maxillary sinus pneumatization does not quantify local bone availability. Although root–sinus relationships and skeletal anchorage are relevant clinical considerations [4,5,7], the present findings cannot establish biomechanical resistance during tooth movement, root proximity to the sinus, or miniscrew stability. Treatment outcomes and nasal function were not assessed; these comparisons therefore provide no direct basis for treatment-specific or CBCT acquisition recommendations.

The nominal finding for the paradoxical middle turbinate should be interpreted in this context. A paradoxical middle turbinate can alter the middle-meatal corridor, whereas the supreme turbinate lies in the posterior superior nasal cavity and may be related to posterior ethmoid drainage [9,10,30,31,32]. In this study, the paradoxical middle turbinate was most frequent in the normodivergent group. The supreme turbinate showed a visually different prevalence distribution, but its omnibus GEE result did not reach the nominal significance threshold (*p* = 0.0597). These frequencies may depend on image orientation, the minimum morphologic extent required for classification, and whether data are reported by side or participant. Because the result for the paradoxical middle turbinate did not survive FDR correction and the pairwise CIs were wide, attributing either distribution to a specific vertical developmental mechanism would be premature.

Sphenoid-region findings included pneumatization patterns and variations of the adjacent neurovascular canals. Pneumatization of the anterior clinoid process, greater wing, or pterygoid process can bring the sphenoid sinus into proximity to the optic canal, internal carotid artery, vidian canal, and maxillary nerve canal. Onodi cells may further modify the posterior ethmoid–optic nerve relationship [33,34,35,36,37,38]. No estimable sphenoid- or canal-related omnibus GEE effect was significant. For carotid canal dehiscence and sphenoid sinus hypoplasia, however, zero-event groups caused separation and prevented valid omnibus GEE comparisons. These non-estimable results reflect limitations of the observed event distribution and cannot be interpreted as evidence of biological homogeneity or anatomical equivalence.

Several features strengthen this study. All participants had skeletal and dental Class I relationships and no previous orthodontic treatment. The detailed eligibility screening deliberately reduced sagittal and treatment-related heterogeneity to improve comparability between vertical groups; the small proportion of eligible records reflects these combined criteria. This restriction is also relevant to findings by Azizia et al., who reported significantly greater maxillary sinus width and depth in Class I than Class III adults, although sinus volume, surface area, and height did not differ significantly across the three skeletal classes [46]. Vertical grouping required agreement between GoGn–SN and FMA rather than relying on a single angle. All CBCT examinations were obtained with one scanner, a 0.25 mm isotropic voxel, and the same reconstruction interval, eliminating cross-scanner imbalance. Bilateral findings were analyzed using GEE to avoid treating two sides from one participant as independent observations. The study also used explicit definitions, including confirmation of canal dehiscence in two orthogonal planes, and the complete sample was reassessed after 1 month with high intraobserver agreement (Cohen’s κ = 0.971–1.000). Finally, FDR correction provided a more conservative interpretation of the 20 estimable omnibus tests among the 22 side-level outcomes.

Some limitations should nevertheless be considered. This was a retrospective, single-center archive study, and the examinations had been obtained for heterogeneous clinical indications. Residual confounding related to clinical indications cannot be excluded; the indication was unavailable for three participants. Although the screening flow was reconstructed at two levels, counts were not available for each individual exclusion category. The modest group sizes and limited event counts restrict the exclusion of small-to-moderate associations, particularly for rare variants; nonsignificant findings do not establish anatomical equivalence. Carotid canal dehiscence and sphenoid sinus hypoplasia contained zero-event groups, precluding finite GEE log-odds and Wald estimates, and several other findings produced wide pairwise CIs. The narrow age range improves developmental comparability but limits generalization to other age groups. Because the sample comprised only untreated adults with skeletal and dental Class I relationships, the findings cannot be generalized to patients with skeletal Class II or Class III relationships or to the broader orthodontic population. CBCT has limited soft-tissue contrast. Limited spatial resolution and partial-volume averaging can impair visualization of thin bone [47]; with the 0.25 mm voxels used here, very thin canal walls, including those <0.5 mm thick, may therefore have been misclassified as dehiscent. Although loss of cortical continuity was required in at least two orthogonal planes, this criterion could not exclude imaging-related misclassification; histologic confirmation was not available. In addition, no symptom scores, nasal airflow measurements, endoscopic findings, medical CT confirmation, sinus-volume segmentation, or treatment outcomes were available. Smoking history and allergic rhinitis information could not be retrieved from the archives. Their potential influence on mucosal thickening could therefore not be assessed or controlled.

For findings defined by numerical thresholds or anatomical extent, binary recording did not capture the magnitude of septal displacement, mucosal thickness, or sinus extension. Lower numerical cutoffs could increase recorded prevalence, whereas higher cutoffs could decrease it; their effects on between-group associations cannot be inferred from the binary data. Continuous values were not recorded, precluding sensitivity analyses using alternative cutoffs. Comparisons with other studies should therefore account for differences in operational definitions.

Future studies should use larger multicenter cohorts, prespecified primary outcomes, and harmonized acquisition and reconstruction protocols. Sample-size planning should reflect the prevalence of prespecified outcomes, within-participant correlation, and the intended multiplicity adjustment. Quantitative anatomical measurements and relevant clinical outcomes could be assessed alongside categorical variants.

## 5. Conclusions

In this retrospective study of 87 untreated adults with skeletal and dental Class I relationships, no statistically significant associations were detected between vertical skeletal pattern and the evaluated sinonasal anatomical variations among estimable comparisons after correction for multiple testing. The overall findings remained consistent after adjustment for sex. However, small-to-moderate associations cannot be excluded, particularly for rare variants. Larger studies are warranted to obtain more precise estimates of these associations.

## Figures and Tables

**Table 2:** Participant characteristics according to vertical skeletal pattern.

Characteristic	Hypodivergent (*n =* 27)	Hyperdivergent (*n* = 26)	Normodivergent (*n* = 34)	Statistic	*p*
Age, years: mean (SD); median (range)	21.5 (2.9); 21 (18–29)	21.0 (3.2); 21 (18–29)	20.6 (2.3); 20 (18–27)	1.358 ‡	0.5072
Women, n (%)	18 (66.7)	18 (69.2)	20 (58.8)	0.786 †	0.6750
Men, n (%)	9 (33.3)	8 (30.8)	14 (41.2)		

† Pearson’s chi-square test; ‡ Kruskal–Wallis test (df = 2).

**Table 3:** Participant-level nasal septal findings.

Finding Present	Hypodivergent (*n* = 27)	Hyperdivergent (*n* = 26)	Normodivergent (*n* = 34)	χ^2^	*p*
Nasal septal deviation	24 (88.9)	20 (76.9)	24 (70.6)	2.986	0.2247
Septal spur	10 (37.0)	9 (34.6)	11 (32.4)	0.146	0.9294

Data are n (%); Pearson’s chi-square test (df = 2).

**Table 4:** Side-level prevalence of anatomic variations according to vertical skeletal pattern.

Finding Present	Hypodivergent (54 Sides)	Hyperdivergent (52 Sides)	Normodivergent (68 Sides)	GEE χ^2^	*p*	q
Haller cell	20 (37.0)	26 (50.0)	26 (38.2)	1.502	0.4719	0.7307
Maxillary sinus septum	17 (31.5)	16 (30.8)	35 (51.5)	4.787	0.0913	0.4642
Accessory maxillary ostium	26 (48.1)	32 (61.5)	36 (52.9)	1.419	0.4919	0.7307
Extensive maxillary sinus pneumatization	32 (59.3)	43 (82.7)	41 (60.3)	4.307	0.1161	0.4642
Mucous retention cyst	3 (5.6)	4 (7.7)	6 (8.8)	0.282	0.8683	0.9103
Mucosal thickening >3 mm	11 (20.4)	6 (11.5)	15 (22.1)	1.479	0.4773	0.7307
Paradoxical middle turbinate	14 (25.9)	6 (11.5)	24 (35.3)	6.199	0.0451	0.4642
Concha bullosa	41 (75.9)	34 (65.4)	52 (76.5)	1.341	0.5115	0.7307
Supreme turbinate	16 (29.6)	2 (3.8)	11 (16.2)	5.637	0.0597	0.4642
Optic canal protrusion	50 (92.6)	43 (82.7)	50 (73.5)	3.479	0.1756	0.5854
Optic canal dehiscence	2 (3.7)	1 (1.9)	5 (7.4)	1.497	0.4730	0.7307
Vidian canal protrusion	27 (50.0)	33 (63.5)	44 (64.7)	1.996	0.3686	0.7307
Vidian canal dehiscence	11 (20.4)	8 (15.4)	15 (22.1)	0.573	0.7507	0.9103
Maxillary nerve canal protrusion	26 (48.1)	29 (55.8)	37 (54.4)	0.485	0.7848	0.9103
Maxillary nerve canal dehiscence	9 (16.7)	7 (13.5)	8 (11.8)	0.373	0.8300	0.9103
Carotid canal protrusion	33 (61.1)	39 (75.0)	42 (61.8)	1.871	0.3925	0.7307
Carotid canal dehiscence	2 (3.7)	0 (0.0)	2 (2.9)	NE	NE	NE
Anterior clinoid process pneumatization	15 (27.8)	13 (25.0)	20 (29.4)	0.188	0.9103	0.9103
Greater wing pneumatization	35 (64.8)	38 (73.1)	39 (57.4)	1.967	0.3740	0.7307
Pterygoid process pneumatization	31 (57.4)	33 (63.5)	37 (54.4)	0.648	0.7232	0.9103
Onodi cell	6 (11.1)	3 (5.8)	15 (22.1)	4.751	0.0930	0.4642
Sphenoid sinus hypoplasia	0 (0.0)	1 (1.9)	0 (0.0)	NE	NE	NE

Data are n (%) of sides. GEE accounts for two sides clustered within each participant. q values are Benjamini–Hochberg FDR-adjusted across the 20 estimable omnibus GEE tests. NE, not estimable: zero-event groups caused separation and prevented a valid omnibus Wald comparison in the specified three-group GEE model. No GEE *p*-value or q-value is reported for these outcomes; NE does not denote a nonsignificant result. GEE, generalized estimating equations; df, degrees of freedom. Estimable omnibus group effects were evaluated with Wald chi-square tests with 2 df.

## Data Availability

The data presented in this study are available on request from the corresponding author. The data are not publicly available due to privacy or ethical restrictions.

## Supplementary Materials

The following supporting information can be downloaded at: https://www.mdpi.com/article/10.3390/diagnostics16213534/s1, Table S1: Right–left comparisons in the hypodivergent group; Table S2: Right–left comparisons in the hyperdivergent group; Table S3: Right–left comparisons in the normodivergent group; Table S4: Pairwise odds ratios derived from the side-level logistic GEE models. Table S5: Sex-adjusted vertical-group comparisons; Table S6: Sex coefficients from the adjusted models.

## Abbreviations

The following abbreviations are used in this manuscript:

FDR	False-discovery-rate
GEE	Generalized estimating equation
CBCT	Cone-beam computed tomography
FMA	Frankfort–mandibular plane
SARME	Surgically assisted rapid maxillary expansion
