# Supplementary material for: Sinonasal Anatomic Variations Across Vertical Skeletal Patterns in Skeletal Class I Adults: A Retrospective Cone-Beam Computed Tomography Study
Source: Diagnostics (Basel). 2026 Oct 28;16(21):3534. doi: 10.3390/diagnostics16213534 (PMC13761572; doi:10.3390/diagnostics16213534)
Supplement: Supplementary file 1 [file diagnostics-16-03534-s001.zip › diagnostics-4574454-supplementary.pdf]

Supplementary Tables

Table S1. Right–left comparisons in the hypodivergent group.

Finding present	Right, n (%)	Left, n (%)	Exact McNemar p
Haller cell	10 (37.0)	10 (37.0)	1.0000
Maxillary sinus septum	7 (25.9)	10 (37.0)	0.4531
Accessory maxillary ostium	14 (51.9)	12 (44.4)	0.7266
Extensive maxillary sinus pneumatization	16 (59.3)	16 (59.3)	1.0000
Mucous retention cyst	1 (3.7)	2 (7.4)	1.0000
Mucosal thickening >3 mm	6 (22.2)	5 (18.5)	1.0000
Paradoxical middle turbinate	7 (25.9)	7 (25.9)	1.0000
Concha bullosa	22 (81.5)	19 (70.4)	0.4531
Supreme turbinate	6 (22.2)	10 (37.0)	0.2188
Optic canal protrusion	25 (92.6)	25 (92.6)	1.0000
Optic canal dehiscence	1 (3.7)	1 (3.7)	1.0000
Vidian canal protrusion	13 (48.1)	14 (51.9)	1.0000
Vidian canal dehiscence	6 (22.2)	5 (18.5)	1.0000
Maxillary nerve canal protrusion	13 (48.1)	13 (48.1)	1.0000
Maxillary nerve canal dehiscence	4 (14.8)	5 (18.5)	1.0000
Carotid canal protrusion	18 (66.7)	15 (55.6)	0.2500
Carotid canal dehiscence	1 (3.7)	1 (3.7)	1.0000
Anterior clinoid process pneumatization	7 (25.9)	8 (29.6)	1.0000
Greater wing pneumatization	18 (66.7)	17 (63.0)	1.0000
Pterygoid process pneumatization	15 (55.6)	16 (59.3)	1.0000
Onodi cell	2 (7.4)	4 (14.8)	0.6875
Sphenoid sinus hypoplasia	0 (0.0)	0 (0.0)	1.0000

For comparisons with no discordant pairs, the exact McNemar p-value is reported as 1.0000; this does not establish right–left equivalence.

Table S2. Right–left comparisons in the hyperdivergent group.

Finding present	Right, n (%)	Left, n (%)	Exact McNemar p
Haller cell	12 (46.2)	14 (53.8)	0.6875
Maxillary sinus septum	6 (23.1)	10 (38.5)	0.2891
Accessory maxillary ostium	18 (69.2)	14 (53.8)	0.2891
Extensive maxillary sinus pneumatization	21 (80.8)	22 (84.6)	1.0000
Mucous retention cyst	1 (3.8)	3 (11.5)	0.6250
Mucosal thickening >3 mm	4 (15.4)	2 (7.7)	0.5000
Paradoxical middle turbinate	4 (15.4)	2 (7.7)	0.6250
Concha bullosa	16 (61.5)	18 (69.2)	0.6250
Supreme turbinate	1 (3.8)	1 (3.8)	1.0000
Optic canal protrusion	22 (84.6)	21 (80.8)	1.0000
Optic canal dehiscence	0 (0.0)	1 (3.8)	1.0000
Vidian canal protrusion	16 (61.5)	17 (65.4)	1.0000
Vidian canal dehiscence	3 (11.5)	5 (19.2)	0.6250
Maxillary nerve canal protrusion	17 (65.4)	12 (46.2)	0.1797
Maxillary nerve canal dehiscence	1 (3.8)	6 (23.1)	0.0625
Carotid canal protrusion	21 (80.8)	18 (69.2)	0.3750
Carotid canal dehiscence	0 (0.0)	0 (0.0)	1.0000
Anterior clinoid process pneumatization	6 (23.1)	7 (26.9)	1.0000
Greater wing pneumatization	18 (69.2)	20 (76.9)	0.6250
Pterygoid process pneumatization	15 (57.7)	18 (69.2)	0.3750
Onodi cell	2 (7.7)	1 (3.8)	1.0000
Sphenoid sinus hypoplasia	1 (3.8)	0 (0.0)	1.0000

For comparisons with no discordant pairs, the exact McNemar p-value is reported as 1.0000; this does not establish right–left equivalence.

Table S3. Right–left comparisons in the normodivergent group.

Finding present	Right, n (%)	Left, n (%)	Exact McNemar p
Haller cell	14 (41.2)	12 (35.3)	0.6875
Maxillary sinus septum	17 (50.0)	18 (52.9)	1.0000
Accessory maxillary ostium	16 (47.1)	20 (58.8)	0.2188
Extensive maxillary sinus pneumatization	19 (55.9)	22 (64.7)	0.2500
Mucous retention cyst	3 (8.8)	3 (8.8)	1.0000
Mucosal thickening >3 mm	10 (29.4)	5 (14.7)	0.1250
Paradoxical middle turbinate	14 (41.2)	10 (29.4)	0.2891
Concha bullosa	25 (73.5)	27 (79.4)	0.6250
Supreme turbinate	6 (17.6)	5 (14.7)	1.0000
Optic canal protrusion	25 (73.5)	25 (73.5)	1.0000
Optic canal dehiscence	2 (5.9)	3 (8.8)	1.0000
Vidian canal protrusion	23 (67.6)	21 (61.8)	0.6250
Vidian canal dehiscence	6 (17.6)	9 (26.5)	0.2500
Maxillary nerve canal protrusion	18 (52.9)	19 (55.9)	1.0000
Maxillary nerve canal dehiscence	5 (14.7)	3 (8.8)	0.5000
Carotid canal protrusion	20 (58.8)	22 (64.7)	0.6250
Carotid canal dehiscence	1 (2.9)	1 (2.9)	1.0000
Anterior clinoid process pneumatization	8 (23.5)	12 (35.3)	0.3438
Greater wing pneumatization	19 (55.9)	20 (58.8)	1.0000
Pterygoid process pneumatization	17 (50.0)	20 (58.8)	0.5078
Onodi cell	7 (20.6)	8 (23.5)	1.0000
Sphenoid sinus hypoplasia	0 (0.0)	0 (0.0)	1.0000

For comparisons with no discordant pairs, the exact McNemar p-value is reported as 1.0000; this does not establish right–left equivalence.

Table S4. Pairwise odds ratios derived from the side-level logistic GEE models.

Finding	Hypodivergent vs Normodivergent	Hyperdivergent vs Normodivergent	Hypodivergent vs Hyperdivergent
Haller cell	0.95 (0.39–2.33)	1.62 (0.64–4.05)	0.59 (0.23–1.50)
Maxillary sinus septum	0.43 (0.18–1.07)	0.42 (0.17–1.03)	1.03 (0.40–2.68)
Accessory maxillary ostium	0.83 (0.34–1.99)	1.42 (0.58–3.47)	0.58 (0.23–1.44)
Extensive maxillary sinus pneumatization	0.96 (0.35–2.63)	3.15 (0.97–10.24)	0.30 (0.09–1.06)
Mucous retention cyst	0.61 (0.10–3.82)	0.86 (0.20–3.67)	0.71 (0.12–4.21)
Mucosal thickening >3 mm	0.90 (0.35–2.32)	0.46 (0.13–1.66)	1.96 (0.55–6.95)
Paradoxical middle turbinate	0.64 (0.25–1.65)	0.24 (0.08–0.74)	2.68 (0.81–8.87)
Concha bullosa	0.97 (0.35–2.67)	0.58 (0.21–1.63)	1.67 (0.60–4.64)
Supreme turbinate	2.18 (0.73–6.52)	0.21 (0.02–1.81)	10.53 (1.26–87.72)
Optic canal protrusion	4.50 (0.89–22.65)	1.72 (0.51–5.85)	2.62 (0.46–14.95)
Optic canal dehiscence	0.48 (0.05–5.02)	0.25 (0.02–2.48)	1.96 (0.12–32.19)
Vidian canal protrusion	0.55 (0.22–1.37)	0.95 (0.36–2.49)	0.58 (0.22–1.51)
Vidian canal dehiscence	0.90 (0.29–2.83)	0.64 (0.20–2.07)	1.41 (0.41–4.84)
Maxillary nerve canal protrusion	0.78 (0.32–1.91)	1.06 (0.44–2.51)	0.74 (0.30–1.83)
Maxillary nerve canal dehiscence	1.50 (0.40–5.64)	1.17 (0.32–4.26)	1.29 (0.37–4.49)
Carotid canal protrusion	0.97 (0.37–2.57)	1.86 (0.68–5.06)	0.52 (0.18–1.51)
Carotid canal dehiscence	NE	NE	NE
Anterior clinoid process pneumatization	0.92 (0.37–2.30)	0.80 (0.29–2.19)	1.15 (0.40–3.37)
Greater wing pneumatization	1.37 (0.51–3.66)	2.02 (0.75–5.40)	0.68 (0.23–2.03)
Pterygoid process pneumatization	1.13 (0.44–2.91)	1.46 (0.58–3.64)	0.78 (0.28–2.18)
Onodi cell	0.44 (0.16–1.22)	0.22 (0.04–1.10)	2.04 (0.38–11.10)
Sphenoid sinus hypoplasia	NE	NE	NE

Data are OR (95% CI). An OR > 1 indicates higher odds in the first-named group. Pairwise estimates are secondary and unadjusted; the omnibus group tests and their FDR-adjusted q values are reported in Table 4. NE, not estimable from the reported three-group model. Pairwise ORs were not reported for outcomes whose full GEE model did not yield a complete set of finite group-effect estimates because of zero-event groups. NE does not imply a nonsignificant comparison.

Table S5. Sex-adjusted vertical-group comparisons.

Finding	Hypo vs normo aOR (95% CI)	Hyper vs normo aOR (95% CI)	p	q
Nasal septal deviation	3.23 (0.79–13.18)	1.31 (0.40–4.31)	0.2622	—
Septal spur	1.26 (0.44–3.64)	1.14 (0.39–3.38)	0.9103	—
Haller cell	0.92 (0.38–2.22)	1.55 (0.61–3.96)	0.5220	0.7629
Maxillary sinus septum	0.44 (0.18–1.10)	0.43 (0.18–1.04)	0.1018	0.4073
Accessory maxillary ostium	0.79 (0.33–1.88)	1.35 (0.54–3.35)	0.5143	0.7629
Extensive maxillary sinus pneumatization	1.00 (0.36–2.76)	3.36 (1.02–11.08)	0.1016	0.4073
Mucous retention cyst	0.63 (0.10–3.87)	0.90 (0.21–3.81)	0.8808	0.9257
Mucosal thickening >3 mm	0.97 (0.37–2.55)	0.50 (0.14–1.82)	0.5192	0.7629
Paradoxical middle turbinate	0.66 (0.25–1.75)	0.25 (0.08–0.76)	0.05003	0.4073
Concha bullosa	0.96 (0.35–2.63)	0.57 (0.20–1.61)	0.4985	0.7629
Supreme turbinate	2.12 (0.70–6.36)	0.20 (0.02–1.68)	0.0594	0.4073
Optic canal protrusion	4.38 (0.87–22.13)	1.65 (0.48–5.70)	0.1960	0.6208
Optic canal dehiscence	NE	NE	NE	NE
Vidian canal protrusion	0.56 (0.22–1.44)	0.99 (0.38–2.58)	0.3983	0.7629
Vidian canal dehiscence	0.91 (0.28–2.94)	0.65 (0.20–2.12)	0.7606	0.9221
Maxillary nerve canal protrusion	0.83 (0.31–2.19)	1.17 (0.52–2.65)	0.7765	0.9221
Maxillary nerve canal dehiscence	1.47 (0.37–5.78)	1.13 (0.31–4.18)	0.8513	0.9257
Carotid canal protrusion	1.09 (0.39–3.08)	2.28 (0.81–6.41)	0.2584	0.7013
Carotid canal dehiscence	NE	NE	NE	NE
Anterior clinoid process pneumatization	0.94 (0.38–2.32)	0.82 (0.30–2.22)	0.9257	0.9257
Greater wing pneumatization	1.43 (0.53–3.90)	2.15 (0.80–5.77)	0.3109	0.7383
Pterygoid process pneumatization	1.21 (0.45–3.23)	1.60 (0.64–3.99)	0.6035	0.8190
Onodi cell	0.46 (0.17–1.27)	0.23 (0.05–1.15)	0.1072	0.4073
Sphenoid sinus hypoplasia	NE	NE	NE	NE

aOR, adjusted odds ratio; CI, confidence interval; NE, not estimable. Models contain vertical group and sex (female versus male). Bilateral analyses use logistic GEE with participant clustering, an exchangeable working correlation, and robust covariance; participant-level septal findings use logistic regression with robust covariance. Omnibus group tests have 2 df. Group q-values are Benjamini–Hochberg adjusted across 19 estimable bilateral models; — denotes participant-level tests outside that FDR family. CIs are nominal 95% intervals, not multiplicity-adjusted.

Table S6. Sex coefficients from the adjusted models.

Finding	Female vs male aOR (95% CI)	Sex p	Sex q
Nasal septal deviation	1.82 (0.63–5.26)	0.2654	—
Septal spur	0.74 (0.30–1.84)	0.5151	—
Haller cell	1.60 (0.70–3.64)	0.2631	0.5596
Maxillary sinus septum	0.80 (0.37–1.74)	0.5789	0.6627
Accessory maxillary ostium	1.73 (0.81–3.69)	0.1548	0.5596
Extensive maxillary sinus pneumatization	0.60 (0.23–1.57)	0.2945	0.5596
Mucous retention cyst	0.63 (0.17–2.38)	0.4994	0.6627
Mucosal thickening >3 mm	0.42 (0.17–1.05)	0.0631	0.3305
Paradoxical middle turbinate	0.61 (0.25–1.48)	0.2751	0.5596
Concha bullosa	1.22 (0.50–2.97)	0.6618	0.6986
Supreme turbinate	1.66 (0.55–4.95)	0.3659	0.5794
Optic canal protrusion	1.54 (0.50–4.72)	0.4512	0.6595
Optic canal dehiscence	NE	NE	NE
Vidian canal protrusion	0.67 (0.30–1.50)	0.3286	0.5675
Vidian canal dehiscence	0.90 (0.33–2.43)	0.8281	0.8281
Maxillary nerve canal protrusion	0.38 (0.17–0.84)	0.0170	0.1618
Maxillary nerve canal dehiscence	1.38 (0.42–4.50)	0.5930	0.6627
Carotid canal protrusion	0.22 (0.08–0.60)	0.0029	0.0557
Carotid canal dehiscence	NE	NE	NE
Anterior clinoid process pneumatization	0.80 (0.37–1.73)	0.5748	0.6627
Greater wing pneumatization	0.59 (0.24–1.43)	0.2433	0.5596
Pterygoid process pneumatization	0.46 (0.19–1.07)	0.0696	0.3305
Onodi cell	0.55 (0.21–1.40)	0.2082	0.5596
Sphenoid sinus hypoplasia	NE	NE	NE

aORs compare women with men, adjusted for vertical skeletal-pattern group, using the models described in Table S5. Sex q-values are Benjamini–Hochberg adjusted across the 19 estimable bilateral sex coefficients as a separate family; — denotes participant-level tests outside that family. NE denotes separation, not lack of an association. CIs are nominal 95% intervals. No sex coefficient survived FDR correction.
